# Effects of very low-calorie ketogenic diet on hypothalamic–pituitary–adrenal axis and renin–angiotensin–aldosterone system

**DOI:** 10.1007/s40618-023-02068-6

**Published:** 2023-04-05

**Authors:** L. Barrea, L. Verde, E. Camajani, A. S. Šojat, L. Marina, S. Savastano, A. Colao, M. Caprio, G. Muscogiuri

**Affiliations:** 1 Dipartimento di Scienze Umanistiche, Università Telematica Pegaso, Via Porzio, Centro Direzionale, Isola F2, 80143 Naples, Italy; 2 Department of Clinical Medicine and Surgery, Endocrinology Unit, Centro Italiano per la cura e il Benessere del Paziente con Obesità (C.I.B.O), University Medical School of Naples, Via Sergio Pansini 5, 80131 Naples, Italy; 3 Department of Public Health, Federico II University, Naples, Italy; 4 Department of Human Sciences and Promotion of the Quality of Life, San Raffaele Roma Open University, 00166 Rome, Italy; 5 Department for Obesity, Metabolic and Reproductive Disorders, Clinic for Endocrinology, Diabetes and Metabolic Diseases, University Clinical Centre of Serbia, Belgrade, Serbia; 6 Dipartimento di Medicina Clinica e Chirurgia, Diabetologia ed Andrologia, Unità di Endocrinologia, Università Federico II, Via Sergio Pansini 5, 80131 Naples, Italy; 7 Cattedra Unesco “Educazione Alla Salute E Allo Sviluppo Sostenibile”, University Federico II, Naples, Italy; 8 Laboratory of Cardiovascular Endocrinology, IRCCS San Raffaele Roma, 00166 Rome, Italy

**Keywords:** Cortisol, HPA axis, Stress, Obesity, Diet, Very low-calorie ketogenic diet

## Abstract

**Background:**

The hypothalamic–pituitary–adrenal (HPA) axis is a neuroendocrine system involved in controlling stress responses in humans under physiological and pathological conditions; cortisol is the main hormone produced by the HPA axis. It is known that calorie restriction acts as a stressor and can lead to an increase in cortisol production. Renin–angiotensin–aldosterone system (RAAS) is a complex endocrine network regulating blood pressure and hydrosaline metabolism, whose final hormonal effector is aldosterone. RAAS activation is linked to cardiometabolic diseases, such as heart failure and obesity. Obesity has become a leading worldwide pandemic, associated with serious health outcomes. Calorie restriction represents a pivotal strategy to tackle obesity. On the other hand, it is well known that an increased activity of the HPA may favour visceral adipose tissue expansion, which may jeopardize a successful diet-induced weight loss. Very low-calorie ketogenic diet (VLCKD) is a normoprotein diet with a drastic reduction of the carbohydrate content and total calorie intake. Thanks to its sustained protein content, VLCKD is extremely effective to reduce adipose tissue while preserving lean body mass and resting metabolic rate.

**Purpose:**

The purpose of this narrative review is to gain more insights on the effects of VLCKD on the HPA axis and RAAS, in different phases of weight loss and in different clinical settings.

## Introduction

The purpose of this narrative review is to gain more insights on the effects of very low-calorie ketogenic diet (VLCKD) on the hypothalamic–pituitary–adrenal (HPA) axis and renin–angiotensin–aldosterone-system (RAAS), in different phases of weight loss and in different clinical settings.

**Fig. 1:**
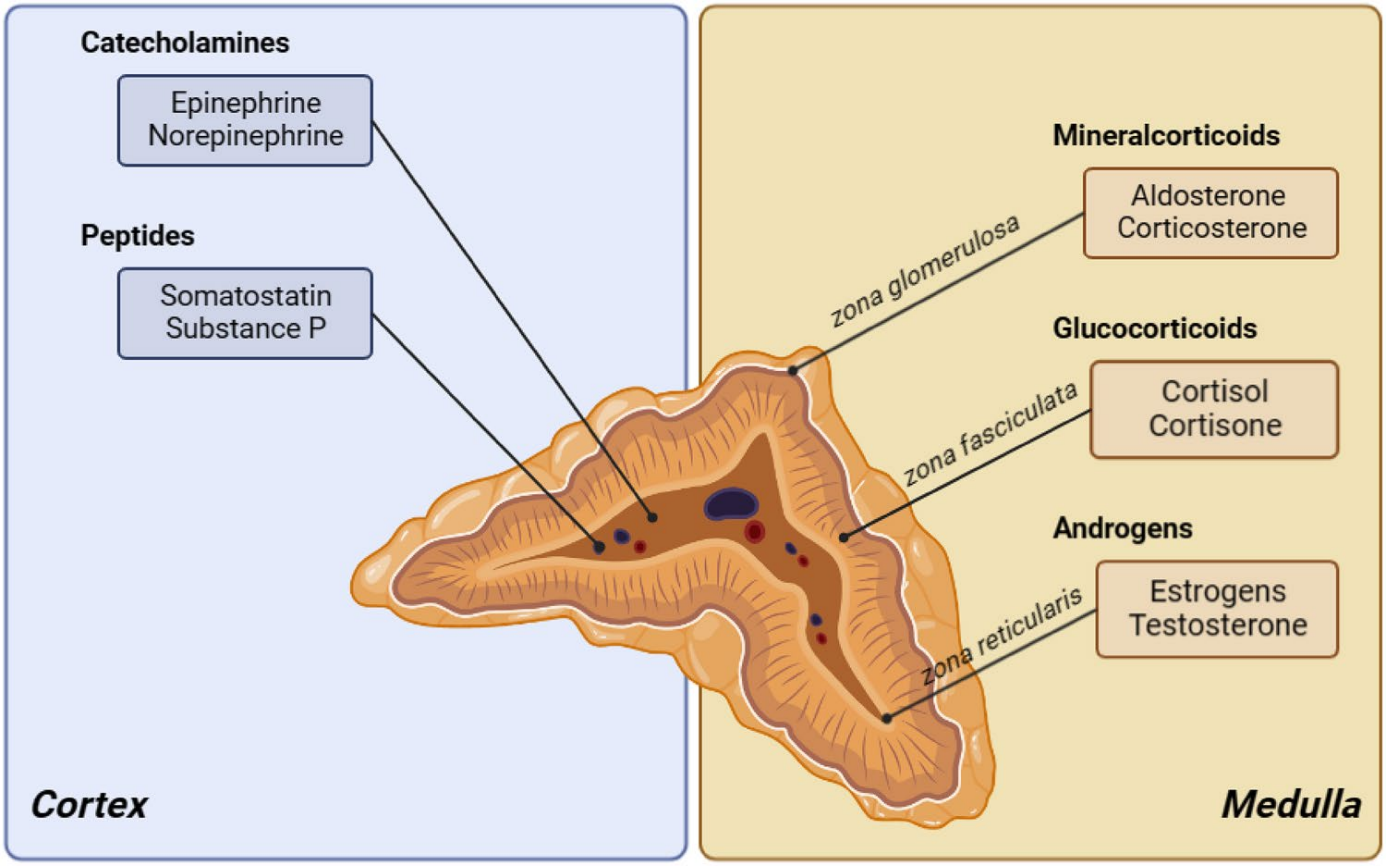
Adrenal gland and the division of the two distinct anatomical areas, with their hormone production

The adrenal gland is a bilateral endocrine organ, located to the superior pool of each kidney, and is composed of two distinct anatomical areas: cortex and medulla [1, 2]. According to Kim and Choi, the adrenal cortex synthesizes steroid hormones from cholesterol through a series of biochemical metabolic pathways [1, 2]. Furthermore, the adrenal cortex consists of three different cortical zones: the glomerulosa zone, secreting mineralocorticoids such as aldosterone, the intermediate fasciculate zone, secreting glucocorticoids such as cortisol, and the innermost reticularis zone, secreting androgens [3, 4], as shown in Fig. 1, while the medullary area produces epinephrine and norepinephrine as part of the sympathetic nervous system [3, 4].

The HPA axis is a critical neurohormonal system that regulates cognitive, metabolic, immunological, and circadian behaviours and responds to internal and external stressors with continuous dynamic equilibration [5, 6]. Any activity, stimulus or situation that causes stress can be regarded as a stressor. Unlike homeostasis, which represents stability through consistency, allostasis is a capability to maintain stability through dynamic change. If the allostatic response is prolonged, inadequate or overstimulated, the reactive processes may lead to maladaptation and organ damage [7]. Glucocorticoids, secreted by the adrenal cortex, participate in this allostasis, control the gene expression for thousands of genes and exert multiple actions by binding to target tissues and activating mineralocorticoid (MR) and glucocorticoid receptors (GR) [8]. Upon reaching the systemic circulation, in healthy individuals, 90% of cortisol is bound to cortisol binding globulin, 5% to albumin leaving around 5–6% in an unbound, active state. The HPA axis activity also depends on glucocorticoid metabolism, clearance and plasma proteins. Glucocorticoids are metabolized irreversibly in the liver with the help of 5α and 5β reductase enzymes. Some conditions, such as obesity, alter the metabolic clearance rate causing an increased glucocorticoid secretion, while maintaining normal plasma levels [9]. Since overt hypercortisolism leads to metabolic manifestations such as visceral fat accumulation, hypertension and diabetes [10], the HPA axis was suggested as a contributor to metabolic dysregulation in obesity. Obesity is also often associated with a broad spectrum of different stressogenic factors, such as infertility, since the women seeking pregnancy are older and heavier than what was observed before [11].

GR are ubiquitously expressed in peripheral tissues and have low affinity and high sensitivity for corticosterone [12]. Some of the many effects of glucocorticoids are to oppose insulin action and stimulate energy turnover between proteins, triglycerides, glycogen and free fuel for mitochondrial oxidation [13, 14], while also having significant effects on cardiovascular tissues, vascular contractility, atherosclerotic process and angiogenesis [15].

Visceral fat represents an important target for glucocorticoid action gene expression [16]. Glucocorticoids induce differentiation of adipocytes leading to insulin resistance and an increase in adiposity [17].

Cortisol action on GR is largely under control by enzyme expression and activity: 11β-hydroxysteroid dehydrogenase type 1 (11β-HSD1) that regenerates active cortisol from inactive cortisone and 11β-hydroxysteroid dehydrogenase type 2 (11β-HSD2) that converts cortisol to cortisone [18]. In obesity, the activity of the adipose 11β-HSD1 is increased. This is associated with the state of chronic inflammation [19] which is a hallmark of obesity, and it was shown that anti-inflammatory treatment can reverse this effect to a certain extent [20]. In animal models, mice-overexpressing adipocyte 11β HSD-1 developed all cortisol-related comorbidities such as diabetes, hypertension, and visceral obesity [21]. On the contrary to adipose tissue, the hepatic 11β-HSD1 activity in obesity is decreased. Tissue-specific dysregulation of cortisol metabolism in human obesity and furthermore, excess liver fat increases glucocorticoid metabolite excretion in urine and can further decrease the hepatic 11β-HSD1 activity [22].

It is well known that neuronal networks that regulate food intake are tightly connected to the HPA axis, expressing a significant effect on appetite-satiety centers [23]. Every stress response is stressor-specific and can vary significantly based on its effect on the organism, how one perceives the stress and the possibility of coping [24].

Functional assessment of the HPA axis in patients with obesity can be done using the usual diagnostic procedures for evaluating patients with suspected hypercortisolism; however, the sensitivity and specificity could be significantly reduced [25]. Despite that, dynamic studies in various settings have been performed to investigate the HPA axis in obesity with regard to chronobiological changes, different types of stimulation or dexamethasone suppression [26]. Interestingly, in adult patients with obesity, there is a normal cortisol daily rhythm, normal adreno-cortico-tropic-hormone (ACTH) levels and either normal or even lower single sample of 24-h cortisol levels [27]. Furthermore, male subjects with obesity with metabolic syndrome, hypertension and/or diabetes were found not to have significant differences in urinary free cortisol, salivary cortisol and post dexamethasone cortisol levels in comparison to the subjects without these disorders [28]. Same was found when all cortisol parameters and the number of features of metabolic syndrome were compared [28].

The modulation of GR activity is influenced by different post-translational alterations such as phosphorylation, ubiquitination or gene polymorphisms [29, 30]. Many of these polymorphisms were investigated in relation to the body mass index (BMI) and other markers of metabolic syndrome [29, 30]. The N363S polymorphism is associated with an increased sensitivity to glucocorticoids, increased insulin response to overnight dexamethasone suppression testing and an increased BMI [30], while the *Bcl*l polymorphism, also connected to enhanced sensitivity to glucocorticoids, was associated with an increased abdominal fat mass. Contrary to this, the ER22/23EK carriers had significantly better metabolic profile than noncarriers [29]. However, despite the vastly explored relationship between obesity and HPA axis, the relationship between food intake and this axis is bidirectional and the data on effects of various diets on HPA adaptation, namely hypercortisolism, and their specific differences still do not offer a unanimous conclusion.

### Renin–angiotensin–aldosterone system, aldosterone, and obesity-related metabolic dysfunctions

Aldosterone is secreted by the glomerulosa zone, under the control of a complex regulatory network, namely the RAAS, potassium plasma concentrations, and, at least in part, the HPA [31].

RAAS has a pivotal role in the regulation of blood pressure, fluid and electrolyte balance and is strictly linked to the pathophysiology of several cardiometabolic diseases, such as heart failure, type 2 diabetes, and obesity [32, 33]. In this endocrine system, angiotensinogen (Agt), mainly produced by liver and adipose tissue, is enzymatically cleaved by renin, which is released into circulation by juxtaglomerular epithelioid cells located in the walls of renal afferent arterioles [34], to Ang I. Ang I is then converted by angiotensin converting enzyme (ACE) in Ang II [35]. Ang II exerts most of its physiological effects mainly through two G-protein-coupled receptors, Ang II type 1 (AT1R) and type 2 (AT2R) receptors, causing vasoconstriction and sodium/fluid retention. Hyperactivation of this pathway can lead to deleterious effects such as hypertension, fibrosis, endothelial dysfunction and inflammation [35]. On the other hand, RAAS is also characterized by a counterregulatory arm whose effects are mediated by ACE2 [extensively reviewed in [28, 36], which converts Ang-II to Ang-1,7 and Ang-1,9. These peptides elicits favourable physiologic effects through AT2R and the Mas receptor (MasR), hence counteracting the ACE1/Ang-II/AT1R arm of the RAAS, thereby determining vasodilation, increase in insulin sensitivity, and anti-inflammatory effects [37]. Aldosterone is the final hormonal effector of the RAAS, exerting its complex biological effects in almost all tissues involved in metabolic homeostasis (i.e. adipose tissue, skeletal muscle, liver, pancreas, etc.) through specific activation of MR [38].

A consistent body of evidence supports the presence of a local RAAS in adipose tissue [39], which is known to represent the major site of Agt production after liver. RAAS activation plays a major role in the regulation of adipocyte function [40]. Remarkably, adipose tissue expresses all components of RAAS necessary to generate vasoactive molecules, angiotensin peptides, and also aldosterone, although at very low concentrations [41].

The obesity state represents a condition characterized by elevated plasma aldosterone levels [42, 43] and increased RAAS activation. RAAS hyperactivation represents a major determinant of obesity complex pathophysiology [44]. Dysfunctional adipose tissue shows increased expression of MR, whose specific activation favours white adipogenesis, and inhibits the browning process, i.e. the acquisition of brown-like characteristics by white adipocytes, a process which carries great promise for protection against obesity and metabolic disorders [38]. Administration of MR antagonists prevents diet-induced obesity through induction of browning of white adipose tissue [45, 46], at least in mice models. A large body of evidence suggests that classical RAAS activation in white adipose tissue displays deleterious effect on insulin sensitivity and inflammation and plays a major role in the development of obesity-related metabolic diseases [47]. Differently, the RAAS counter-regulatory activation has shown favourable effects on adipocyte and metabolic dysfunction: in fact the administration of Ang-1,7 to high-fat diet fed mice improved insulin sensitivity through an increase in Akt phosphorylation in brown adipose tissue [48]. Hence, pharmacological, or nutritional interventions acting on the balance between the classical and counter regulatory arms of the RAAS could potentially display favourable effects in obesity-related metabolic dysfunctions.

On the other hand, weight loss and a reduction in fat mass is able to affect RAAS activation, probably due to a reduction in adipocyte factors (CTRP1, leptin, and other) capable to directly increase aldosterone synthesis by the adrenal gland, independently from renin [49]. In line with this, a recent report showed that bariatric-induced weight loss is able to lower plasma aldosterone concentration, independently of plasma renin activity and sodium excretion [50].

### Diet as a stress

There are clear individual differences in humans in food intake during stressful periods. Age and sex contribute to this diverse response [51]. Even in the absence of hunger, stress tends to precipitate the intake of calorie dense foods, fast food and food rich in sugar, especially in individuals with overweight or obesity. The neural responses in brain regions of patients with obesity associated with motivation, emotion-memory and taste processing are shown to correlate with Homeostatic Model Assessment for Insulin Resistance (HOMA-IR). Alterations in insulin sensitivity can modify and suppress neural pathways associated with stress and food intake [52]. Various diets are available with a wide array of success and different metabolic outcomes. Diet induced weight loss takes place in the setting of increased energy expenditure and a net caloric deficit. Caloric restriction is one of the proven ways to reduce cardiovascular risk [53]. It is linked to a reduction in blood pressure, insulin sensitivity and leads to weight loss [53]. It is well known that the HPA axis is reactive to food intake. Glucocorticoid levels increase immediately after a meal [54]. Nevertheless, the long-term changes in total and tissue specific glucocorticoid metabolism and their relation to weight loss appear to be more complex.

Caloric intake restriction poses a stressor and can lead to cortisol output elevation as a simple manifestation of its physiological role in energy expenditure [55]. Furthermore, Purnell et al. suggest that the increased HPA activity may promote visceral weight regain following a successful diet-induced weight loss. They have shown that metabolic clearance, cortisol production rate and free cortisol do not significantly change in comparison to baseline after 24 weeks of dieting in men with obesity, but in the same group, with further weight loss, cortisol production increased, and the activity of adipose, tissue specific 11β-HSD-1 decreased [56]. In adipose tissue of patients with obesity, the inhibition of 11β-HSD-1 results in tissue-specific cortisol concentration reduction, which was suggested to improve insulin sensitivity in this group [9]. Other studies report similar findings of the unchanged circulating levels of cortisol, cortisone and urinary steroid metabolite ratios and a decreased 11β-HSD-1 activity in adipose tissue [57]. It seems that with a less significant weight loss, the expression of 11β-HSD-1 is mostly unaltered [58], but as the weight loss becomes more significant, this activity decreases. In patients with obesity that were undergoing bariatric surgery, the omental and hepatic 11β-HSD-1 were found to correlate with their BMI and the adipose 11β-HSD-1 was also significantly reduced after significant post-surgical weight loss [59].

Even though BMI is still the most used tool for stratification of obesity, obesity associated risks and the success of an individual weight loss, the body fat mass, the fat free mass and muscle gain can provide further important insight in the quality of the weight loss [60]. Various dietary approaches using low energy density, lower glycaemic index or portion controls have shown to improve body fat percentage and fat mass with no change in fat free mass [61]. In a large cohort of male patients, circulating cortisol levels were negatively associated with weight, BMI, but also waist-to-hip ratio and waist circumference. The longitudinal changes in cortisol levels were also negatively associated with these measures of adiposity [62]. Hair cortisol levels are proposed as one of the ways to measure chronic stress levels, that differ from the urinary, blood or salivary cortisol [63]; however, Larsen et al. found no association between the hair cortisol levels and weight loss maintenance in patients that had achieved a significant weight loss [64].

Manipulation of the macronutrient content is also speculated to alter glucocorticoid metabolism. In animal models, the hepatic 11β-HSD-1 was shown to be reduced in the setting of low or moderate carbohydrate diets in comparison to a high fat diet [65]. The connection between a decreased hepatic and adipose glucocorticoid regeneration and a dietary fat content has not yet been elucidated. The same group has also shown that a low-carbohydrate diet alters cortisol metabolism independently of weight loss, highlighting low-carbohydrate ketogenic diets (LCKDs) as a possible efficient tool for reversing the metabolic consequences of obesity in male patients [66]. Obesity is also an important comorbidity in various endocrinological diseases. An increasing number of patients with obesity are diagnosed with a wide spectrum of hypercortisolism ranging from (possible) autonomous cortisol secretion to overt Cushing’s syndrome and due to a long list of cardio-metabolic comorbidities associated with these conditions, treating these patients requires a personalized and a tailored approach along with lifestyle interventions [67]. Low-carb diets are known to ameliorate all metabolic complications associated with hypercortisolism, including diabetes, insulin resistance, hypertension, and obesity. In patients with both ACTH-dependent and -independent cortisol hypersecretion, it is often advised that the low-carb diet is maintained prior, during and after surgery or medical treatment of the primary disease [68]. So far, the data on the efficacy of VLCKD in treating patients with hypercortisolism are scarce; however, based on the beneficial effects that VLCKD is showing in treating all aforementioned cardio-metabolic comorbidities, Guarnotta et al. speculate that it could be successfully employed in treating these groups of patients [69].

Physical activity goes hand in hand with weight reduction programs and is almost always advised to patients with obesity within their physical limitations. However, exercise can be both a stressor itself and a modifier of stress in relation to the HPA axis [70]. Athletes who performed intense training sessions during the day had suppressed cortisol levels at night. The lowest cortisol levels were found in athletes having the most intense daytime training [70].

In animal models, exercise led to a reduction in GR expression and 11β-HSD-1 in liver and muscles, with unchanged levels of circulating cortisol [71]. However, we cannot determine the full scope of the impact of exercise on the HPA axis and its contribution to diet because of several key modulatory cofactors. Food intake prior exercise is associated with a lower postprandial cortisol elevation, while adversely, performing physical activity after a meal leads to a suppressed cortisol response [72]. Irrespective of the thermal stress in physical activity, hypohydration can significantly modify the hormonal response, manifesting with increased circulating cortisol levels, possibly due to increased internal temperature and a reduction in plasma volume [73]. Also, physical activity during different periods of a day significantly influences cortisol levels and can create more difficulty in interpreting results [74].

Weight loss in response to the low-calorie diet tends to be different in male and female patients with obesity [75, 76]. Energy balance and glucose metabolism are initially distinct between the two in part because of the effect of sex hormones, including not only circulating oestrogen and androgen levels, but also their adipose tissue production and their effect on adipose tissue distribution [77]. Postmenopausal women tend to lose lean body mass with the loss of oestrogen and show an increase in adipose tissue [78]. The number of women with obesity (BMI > 35 kg/m^2^) is almost double in comparison to male patients [79]. Male patients with obesity show a more significant weight loss and improvement in some cardiometabolic factors, while female patients demonstrate a lower regain of weight but have less dietary intervention effect on the cardiometabolic outcomes [80]. On a molecular level, oestrogen has a stimulatory effect on the HPA axis, both centrally and trough increasing cortisol binding globulin, and is opposed by progesterone depending on the phase of the menstrual cycle and age, while androgens down-regulate the stress induced and basal glucocorticoid levels [81]. The relationship between obesity, testosterone and steroidogenesis was investigated in various studies. Although it is well known that obesity is associated with hypogonadism [82] and weight loss promotes testosterone increase, the data on different diet regimes in relation to testosterone changes are still a matter of debate. Testosterone influences lipid, protein and carbohydrate metabolism, and lower testosterone levels favour pluripotent stem cell conversion into adipocytes while leptin itself inhibits testosterone secretion from the Leydig cells [83]. In a recent meta-analysis of 7 studies authors aimed to evaluate the potential effect of ketogenic diets (KDs) on testosterone levels. Of note, the most evident testosterone increase was found in patients with VLCKD. The authors suggest a multifactorial physiological mechanism for this, including a cholesterol intake increase, low fiber intake and alterations in glucose and insulin homeostasis [84].

Individuals with obesity often have a very hard time maintaining the newly reduced weight, even after a successful initial weight loss. Poor appetite control can sometimes be exacerbated by a diet with patients not being able to achieve the significant metabolic and psychological benefits of weight loss. Various studies have shown that dieting and food restriction can lead to anxiety, depression, or irritability [85]. For example, individual behavioural vulnerability can significantly impair the success of a low-energy diet-based weight loss program, highlighting the importance of the individualized approach in obesity management [80]. Restrained eating, a term reflecting an individual struggle to control food intake and weight [86] can pose as one of the stressogenic factors during weight loss. Elevated salivary cortisol levels were found to positively correlate with the level of self-reported dietary restraint in premenopausal women [87]. Furthermore, when taking in consideration other factors, individual differences in cognitive restraint such as body image perception, appearance beliefs and dissatisfaction also emerged as important parameters in the level of stress related to dietary restraint and cortisol levels [88]. In addition, monitoring calories increases perceived stress irrespective of calorie restriction [55]. Obesity has become a leading worldwide pandemic, associated with serious outcomes [89]. Despite all the variables mentioned in this article, the fact that cortisol levels were shown to be lower in patients with obesity, with some studies showing a U-shaped relationship with BMI across the weight spectrum [90] could mean that the subtle hypercortisolism shown in weight loss, especially VLCKD, could not only be a useful physiological consequence, but also a sign of the HPA axis reactivation or return to normal state.

### Mechanisms of action of the ketogenic diet on energy metabolism and muscle mass

**Fig. 2:**
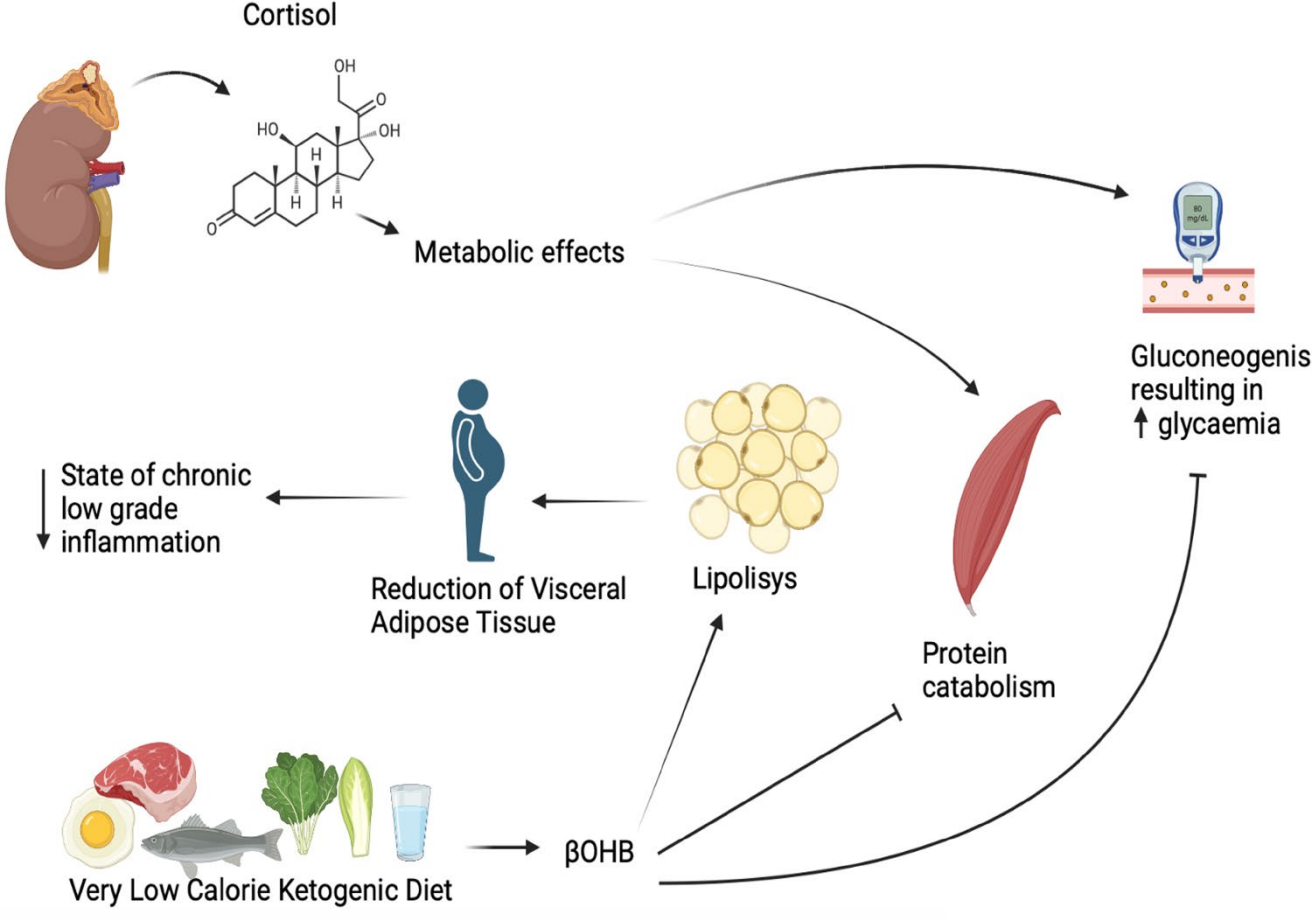
Short-term positive mechanisms of action of the ketogenic diet on energy metabolism and muscle mass

KD is a normoprotein diet with a drastic reduction of the carbohydrate content (approximately between 30 and 50 g/day); depending on the calorie content, KD can be defined as a high fat diet (with a fat content of approximately 60–70%), a LCKD, with a fat content > 30–40 g/day, or a VLCKD, with a fat content < 30–40 g/day [91, 92]. Thanks to their sustained protein content, KD is extremely effective to reduce adipose tissue while preserving lean mass, as reported in Fig. 2.

Importantly, β-hydroxybutyrate (βHB) has also been shown to exert anticatabolic effects on human skeletal muscle [93]. In fact, as reported by Barrea et al., KDs have a favourable impact on lean body mass preservation [94]. As reported by Basolo et al. lean body mass is the main determinant of resting metabolic rate, accounting for ~ 70% [95]. Therefore, it is extremely important, in a very low-calorie diet, to preserve lean mass to maintain resting metabolic rate. Moreover, in a recent pilot study by Camajani et al., the efficacy of a VLCKD on fat free mass, basal metabolic rate and body cell mass was evaluated: 12 patients were enrolled in the control group that underwent only VLCKD and 12 patients were instead enrolled in the experimental group that received the same diet in combination with interval training [96]. At the end of the 6-weeks study, it was seen that the experimental group preserved fat free mass, basal metabolic rate and body cell mass to a greater extent [96].

In the pilot study conducted by Merra et al., it has been demonstrated that VLCKD was highly effective in terms of body weight reduction without inducing lean body mass loss [97]. According to Barrea et al., the preservation of muscle mass, which is positively associated with muscle strength, has been included among the benefits of VLCKD due to the synergistic effects exerted by the reduction in visceral adipose tissue and obesity-related pro-inflammatory status [98]. In fact, in their study, the authors showed that at the end of a 45-days VLCKD protocol, there was an increase in muscle strength (∆ + 17.4 ± 13.2%; *p* = 0.001) [98].

### Activation of the HPA axis during a ketogenic diet

The HPA axis is a neuroendocrine system involved in maintaining homeostasis in humans under physiological conditions and stress, and cortisol is the major hormone of the HPA axis. In obesity, calorie restriction is a good strategy to reduce visceral adipose tissue. In this respect, however, it is well known that dieting is a stress factor for the individual, resulting in negative consequences on body composition and energy metabolism reducing the free fat mass, which is an important site for glucose uptake. Furthermore, studies from the last decade have shown that the KD alters the hormonal balance through changes in the production of metabolic regulatory hormones, such as cortisol [99]. In fact, as reported by Thio, during a KD there is an increasing of serum cortisol levels, in rats [100]. In fact, in the study by Thio and colleagues, rats undergoing KD for 2 weeks had tenfold higher mid-day serum βHB levels and 30% lower glucose levels than rats undergoing standard diet. The elevated βHB levels indicated that KD produced ketosis. The reduction in glucose was expected, as low-carbohydrate diets can lower blood glucose in humans. Rats subjected to KD also had slightly increased cortisol levels (*p* < 0.05) [100].

In a study by Ryan et al., it was demonstrated that a nutritional manipulation characterized by a relative depletion of dietary carbohydrates, thereby inducing nutritional ketosis, acutely and chronically activated the HPA axis [101]. Male rats and mice maintained on a KD exhibited canonical markers of chronic stress, including increased basal and stress-evoked plasma corticosterone, increased adrenal sensitivity to adrenocorticotropin hormone, and thymic atrophy, an indicator of chronic glucocorticoid exposure.

In subjects with obesity, caloric restriction is a valid strategy to reduce visceral adipose tissue. A recent study was carried out by Polito et al. to assess the effects of a VLCKD for weight loss on the sympathetic nervous system and HPA axis, through evaluation of salivary cortisol and galvanic skin response levels [102]. Thirty male subjects with obesity were recruited and assessed before and after 8 weeks of VLCKD intervention to evaluate body composition and biochemical parameters. Salivary cortisol levels and galvanic skin response significantly decreased after dietary treatment; in addition, body composition and biochemical features were ameliorated. They concluded that a VLCKD had a short-term positive effect on the sympathetic nervous system and HPA axes regulating salivary cortisol levels, despite the effect observed in preclinical studies [102]. Despite VLCKD was associated with hyperactivation of the HPA axis as other nutritional protocols do and that is usually associated with fat-free muscle loss, they interestingly found that VLCKD was associated with an increase of fat free mass, probably due to the trophic effects of ketone bodies on muscle mass. Since muscle mass is important for glucose uptake in glucose metabolism, VLCKD could potentially become a promising nutritional approach, mostly in subjects with obesity and glucose metabolism derangements.

VLCKD, thanks to the dramatic reduction of the exogenous carbohydrate content and calories, determines physiological nutritional ketosis: the ketone bodies produced through lipolysis, and especially βHB, have an anti-proteolytic effect, preserving muscle mass.

In addition, there will be a decrease in adipose tissue, particularly visceral adipose tissue, with a reduction in the pro-inflammatory cytokines, corresponding reduction in the chronic low-grade inflammatory state, characteristic of subjects with obesity.

The VLCKD protocol is medicalized and well standardized; the nutritional ketosis phase, and hence the consequent reduction in carbohydrates, can only be prolonged for 12 weeks [100]. Thereafter, there will be a progressive increase in carbohydrate and calorie content, which will not activate stressogenic compensatory hormonal responses that would lead to the consequent increase in cortisol.

### Potential effects of VLCKD on the RAAS

The valuable effects of VLCKD in the rapid reduction of ectopic and visceral fat displays consistent favourable effects on the major risk factors for cardiovascular diseases [22, 103]. Pioneering studies by Blackburn have demonstrated marked effects of VLCKD in the reduction of body weight, together with a significant decrease in blood pressure, fasting glucose and triglyceride plasma levels [104]. VLCKD was shown to be more effective in blood pressure lowering than a combined intervention based on a classical hypocaloric diet combined with orlistat treatment [105]. Such effect is probably linked to the increased natriuresis associated with ketone bodies urinary excretion. A recent meta-analysis of 20 studies found out a modest but significant increase in serum sodium in subject following a VLCKD [106], probably related to the important water loss occurring during the first phases of ketosis. In this context, it is important to keep in mind that a careful supplementation in minerals, including sodium, potassium, calcium, and magnesium, as well as a proper water intake, are mandatory in order to avoid potential side effects due to alteration of hydrosaline metabolism. It appears evident that VLCKD necessarily elicits rapid RAAS responses due to a different salt and water handling during nutritional ketosis. In this context, a very recent report demonstrated that a KD, with or without supplementation in ketone esters, markedly increases aldosterone plasma levels without worsening cardiometabolic risk factors [107]. Importantly, aldosterone plasma concentrations were inversely related to renin, suggesting a renin-independent activation of aldosterone production. Moreover, ketone plasma levels were positively correlated to aldosterone, suggesting a potential novel role of ketones on aldosterone production by the adrenal gland. Importantly, this substantial increase in aldosterone did not determine any adverse effect in cardiometabolic risk factors in patients following a KD, probably due to the well-known cardioprotective effects of ketone bodies [108]. However, the effects of VLCKD on all components of the RAAS still need to be clarified.

A recent study explored the differences existing in RAAS regulation in murine adipose tissue under obesogenic and ketogenic nutritional regimens [109]. The authors tested the hypothesis that the favourable vascular effects of KD were strictly linked to an increased expression in the components of the counterregulatory arm of the RAS. Interestingly, they demonstrated that KD shifted RAAS profile to the counterregulatory arm, whereas an obesogenic nutritional regimen up-regulated the expression of ACE1/Ang-II/AT1R in adipose tissue. These data suggest that VLCKD may directly affect RAAS regulation at different levels, in view of its significant impact on adipose tissue metabolism, fluid/salt regulation, appetite and thirst regulation, natriuresis, etc., potentially counteracting the adverse cardiometabolic consequences of RAAS dysregulation in obesity. The mechanisms underlying these effects are still unclear. Ketone bodies could display powerful effects both on aldosterone secretion by adrenal cells, both on the expression and function of RAAS peptides. Caloric restriction, which shares with RAAS blockade similar effects on longevity [45], could also play a substantial role in the RAAS adaptation to a completely different dietary regimen. However, this hypothesis needs further studies, both in preclinical models exposed to KD, and in patients with obesity undergoing a VLCKD.

## Conclusions

There is a strong relationship between obesity, stress, responses to low calorie diets, weight loss, the HPA axis and the RAAS, which indeed play a key role in short- and long-term metabolic adaptation to a very low-calorie diet. VLCKD represents a valuable nutritional strategy to tackle obesity, inducing a rapid and effective loss of adipose tissue. Its potential impact in the adaptation of the HPA axis and RAAS to a novel metabolic, hormonal, cardiovascular and psychological status, has been poorly addressed so far, and requires ad hoc studies, to understand the effects of VLCKD on adrenal function. VLCKD could display favourable effects against stress-induced hypercortisolism and has been shown to directly increase aldosterone production by the adrenal glands, without any detrimental effect on cardiovascular risk. Importantly, due to a significant loss of visceral and subcutaneous fat, VLCKD may strongly affects the peripheral metabolism of steroid hormones by adipose tissue, with subsequent important impact on cortisol effects on central and peripheral tissues. More studies are deemed necessary in this regard, in order to better define precision nutrition strategies, optimally adapting to the hormonal changes related to weight loss, to maintain the novel metabolic status and avoid weight regain.

## Data Availability

Data sharing not applicable to this article as no datasets were generated or analysed during the current study.

## Abbreviations

HPA: Hypothalamic–pituitary–adrenal
MR: Mineralocorticoid receptor
GR: Glucocorticoid receptors
11β-HSD1: 11β-Hydroxysteroid dehydrogenase type 1
11β-HSD2: 11β-Hydroxysteroid dehydrogenase type 2
ACTH: Adreno-cortico-tropic-hormone
BMI: Body mass index
HOMA-IR: Homeostatic model assessment for insulin resistance
KD: Ketogenic diet
BHB: β-Hydroxybutyrate
RMR: Resting metabolic rate
VLCKD: Very low-calorie ketogenic diet
Ang: Angiotensin
Agt: Angiotensinogen
ACE: Angiotensin-converting enzyme
AT1R: Angiotensin II type 1 receptor
AT2R: Angiotensin II type 2 receptor
